# Targeting Strategies for Aberrant Lipid Metabolism Reprogramming and the Immune Microenvironment in Esophageal Cancer: A Review

**DOI:** 10.1155/2022/4257359

**Published:** 2022-09-05

**Authors:** Meng-Ying Cui, Xing Yi, Zhen-Zhen Cao, Dan-Xia Zhu, Jun Wu

**Affiliations:** Department of Oncology, Third Affiliated Hospital of Soochow University, Changzhou, Jiangsu, China

## Abstract

Esophageal cancer is of high importance to occurrence, development, and treatment resistance. As evidenced by recent studies, pathways (e.g., Wnt/*β*-catenin, AMPK, and Hippo) are critical to the proliferation, differentiation, and self-renewal of esophageal cancer. In addition, the above pathways play a certain role in regulating esophageal cancer and act as potential therapeutic targets. Over the past few years, the function of lipid metabolism in controlling tumor cells and immune cells has aroused extensive attention. It has been reported that there are intricate interactions between lipid metabolism reprogramming between immune and esophageal cancer cells, whereas molecular mechanisms should be studied in depth. Immune cells have been commonly recognized as a vital player in the esophageal cancer microenvironment, having complex crosstalk with cancer cells. It is increasingly evidenced that the function of immune cells in the tumor microenvironment (TME) is significantly correlated with abnormal lipid metabolism. In this review, the latest findings in lipid metabolism reprogramming in TME are summarized, and the above findings are linked to esophageal cancer progression. Aberrant lipid metabolism and associated signaling pathways are likely to serve as a novel strategy to treat esophageal cancer through lipid metabolism reprogramming.

## 1. Introduction

The incidence of esophageal cancer (EC) ranks seventh among all malignant tumors, and there are significant geographical differences in the incidence of esophageal cancer, with rare incidence in developed nations (e.g., Europe and the United States), as indicated by the epidemiological statistics in 2018. The incidence of EC has been extremely high in developing nations [1]. Esophageal cancer primarily has two pathological types, including esophageal squamous cell carcinoma (ESCC) and esophageal adenocarcinoma (EAC). Squamous cell carcinoma has been more commonly diagnosed in males, with a relationship to smoking and alcohol abuse, while adenocarcinoma is correlated with Barrett's esophagus, gastroesophageal reflux, and hiatal hernia [2, 3]. EAC has been common in developed nations, while ESCC is more commonly detected in Asia, Africa, and other developing nations. In addition, China is a high incidence area of esophageal cancer, and there is a significant gender difference in the incidence of esophageal cancer, with male incidence significantly higher than female incidence [4]. The surgical removal of a lesion is considered a highly effective way to cure esophageal cancer, whereas the early symptoms are not typical. Most patients are diagnosed with the disease at the middle and advanced stages, thus missing the opportunity for surgical treatment. Accordingly, radiotherapy, chemotherapy, targeted therapy, immunotherapy, and other comprehensive therapies for the treatment of esophageal cancer are found to play a major role, whereas the overall survival rate of esophageal cancer remains not ideal [5]. As indicated by the latest investigation, significant changes have occurred in the metabolism of a wide range of nutrients in tumor cells, among which lipid metabolism reprogramming has aroused rising attention since it can promote tumorigenesis and metastasis. As the regulatory mechanisms of lipid metabolism have not been comprehensively clarified, the application of targeted lipid metabolism in tumor treatment has emerged. Furthermore, tumor cells interact with immune cells, thus having an effect on the microenvironment of lipid metabolism.

At present, esophageal cancer arises from the action of a defective factor (e.g., a diet, an environment, and a gene), thus leading to body metabolism and the change of cells [6]. As confirmed by existing studies, esophageal cancer are multigene mutations in somatic cell levels, which are essentially correlated with the genetic susceptibility of biological individuals. Moreover, cancer cells are gradually produced in an external environment (e.g., diets, nutrition, and viruses). Subsequently, numerous esophageal cancer-free genes and susceptibility genes have been discovered, and most of them are concentrated in association, lipid metabolism, and vitamin synthesis-related gene regions [7]. Under physiological conditions, saccharides, lipids, and amino acid metabolism lay a basis for the body's normal life activities. However, in patients suffering from esophageal cancer, the amount of energy demand increases since tumor cells are rapidly proliferated, thus resulting in the metabolism of tumor tissue and normal tissue, which is significantly different from [8]. Existing studies have found key enzymes that play a certain role in lipid metabolism, which can increase the incidence of esophageal cancer and have a certain effect on cancer [9, 10]. According to epidemiological studies, the risk of malignant tumors in obese populations includes an elevated risk of esophageal squamous cell, which reveals that fatty acid metabolism is critical to the development of tumors [10–12]. Fatty acids have been found as the main energy source of mammals for the normal growth and metabolism of cells. Fatty acids can be broken down into carbon dioxide and water in vivo, and a considerable amount of energy is released in the form of ATP and exploited by the organism [13]. However, the process of fatty acid oxidation of esophageal cancer and the synthesis of endogenous fatty acids remain unclear. The specific mechanisms of the above fatty acid metabolism, which play a certain role in regulating esophageal squamous cell carcinoma, have been rarely known. Besides, the role played by lipid metabolism in regulating immune cells has aroused extensive attention. Furthermore, immunocytes are critical to an esophageal cancer microenvironment and exhibit complex crosstalk with cancer cells. Thus, the key pathway to the identification of esophageal cancer's metabolism can significantly explore the occurrence of the esophagus, and it can present novel insights into the diagnosis and treatment of esophageal cancer. It is desirable to develop ESCC new biological markers and immune environment drug resistance mechanisms and lay a theoretical basis for targeted treatment.

## 2. Introduction to Esophageal Cancer

Esophageal cancer refers to the eighth most common type of cancer worldwide and the sixth leading cause of cancer death. It is characterized by high mortality, poor prognosis at diagnosis, and geographical variation. This type of cancer includes two primary malignancies, including ESCC and EAC. While ESCC remains the most common type worldwide, EAC is rapidly becoming the most common type in developed nations. Adenocarcinoma and squamous cell carcinoma are recognized as two different tumor types. ESCC is significantly correlated with the life and environmental factors (e.g., drinking and smoking, hot drinking, as well as taking nitrosamines). In addition, the deficiency of micronutrients (e.g., vitamin C, vitamin E, and folic acid) is correlated with the development of squamous cell carcinoma of the esophagus [6]. The above factors may change the genomic pattern of genes and epigenetics. On the whole, the progression of ESCC consists of five stages, that is, simple epithelial hyperplasia, atypical hyperplasia, preinvasion cancer, invasive carcinoma, and metastatic carcinoma. Stunting has been found as a crucial step in the development of ESCC. As indicated by histological analysis, atypical hyperplasia is characterized by basal cell hyperplasia with irregular epithelial stratification and increased normal and abnormal mitosis. Moreover, atypical hyperplasia is manifested as enlarged nucleoli, keratosis of individual cells, loss or decrease in cell cohesion, and loss of cell polarity before malignant transformation [14].

Barrett's esophagus (BE), characterized by the presence of a columnar mucosa of the esophagus, is considered a complication of gastroesophageal reflux disease (GERD) and a precancerous lesion to EAC [15, 16]. Over the past few years, the incidence of EAC has increased rapidly worldwide, especially in developed nations, which probably arises from an increase in obesity and a decrease in *Helicobacter pylori* infection [17]. For esophageal adenocarcinoma, risk factors include BE, gastroesophageal reflux disease, obesity, and tobacco consumption. The development of BE has been the only known risk factor of EAC. The EAC appears to emerge via a metaplasia-atypicalhyperplasia-cancer sequence in which Barrett's metaplasia can develop into low-grade atypical hyperplasia, into high-grade atypical hyperplasia, and finally into intramucosal carcinoma and invasive carcinoma [14]. As revealed by the latest research, ESCC and EAC have different cell sources. It is noteworthy that some of the above models suggest that the malignant transformation of stem cells/progenitor cells leads to the sequential development of tumors [18–20].

Lipid metabolism plays a vital role in the occurrence and development of tumors, either esophageal squamous cell carcinoma or esophageal adenocarcinoma. As indicated by the findings of Shawn A, dietary intake of fish/shellfish, particularly nonfried and high N-3 fatty acids and long-chain N-3 polyunsaturated fatty acids might increase the risk of developing esophageal adenocarcinoma due to its relatively high N-3 unsaturated fatty acid content. Long-chain N-3 PUFAs would be the precursors to anti-inflammatory lipid mediators that inhibit angiogenesis and induce apoptosis. As opposed to the above finding, arachidonic acid of long-chain N-6 PUFA would be a precursor to pro-inflammatory lipid mediators [21, 22]. As demonstrated by Odera et al., NRF2/ACSS2 axis and ethanol exposure could facilitate OXPHOS and lipid synthesis in ESCC cells, thus leading to metabolic reprogramming and enhanced invasiveness. Milada et al. found that FA spectra changed in plasma phosphatidylcholine in esophageal squamous cell carcinoma patients, which might be correlated with abnormal fatty acid metabolism in cancer (e.g., altered de novo fatty acid synthesis, *β*-oxidation, desaturation, and extension). The above changes may be correlated with the prominent functioning of neoadjuvant chemotherapy [23].

### 2.1. The Involvement of Dietary Fats in the Onset of Esophageal Cancer

Glycerol and fatty acids, which are substances including polyunsaturated fatty acids (PUFAs), monounsaturated fatty acids (MUFAs), and saturated fatty acids (SFAs), compensate for the triglyceride that makes up natural dietary fat [24]. On the one hand, fatty acids can promote cancer aggressiveness, but on the other hand, they have been identified as potential targets for anticancer metabolic therapy [25]. To the best of our knowledge, food consumption and nutritional status are being acknowledged as important factors in the treatment of esophageal cancer. Excess adipose tissue resulting from high-fat intake releases more tumorigenic adipokines. Leptin adipokines, for instance, have been demonstrated to promote the proliferation and survival of EAC cell lines in vitro [26]. Ma et al.'s study showed that a high-fat diet can boost the activity of peroxisome proliferator-activated receptor gamma (PPARG) in gastroesophageal cells, and upregulation of PPARG can increase the de novo synthesis of fatty acids, phospholipids, and sphingolipids and promote the occurrence and development of esophageal adenocarcinoma [27]. Esophageal adenocarcinoma (EAC) and esophagogastric junction adenocarcinoma (EGJAC) have been reported to be positively related to meat consumption and high-fat diets [28]. At the same time, dietary fat and specific intake of SFAs and PUFAs were associated with an increased risk of esophageal adenocarcinoma, while substantially inversely linked with esophageal carcinoma squamous cell type [29]. After further stratification by lifestyle exposure factors, Hu et al. found that even-chain unsaturated fatty acid (EC-UFA) patterns were associated with an increased ESCC risk, whereas the N-3long-chain polyunsaturated fatty acid (N-3 LC-PUFA) pattern intake was associated with a reduced risk of ESCC. However, no significant associations were observed with MLC-SFA or SFA patterns among the study subjects [30].

### 2.2. Reprogramming of Lipid Metabolism in Esophageal Cancer

In malignancies, the production of endogenous fats and the consumption of exogenous lipids can satisfy the high demand of cancer cells for lipids. They are highly coordinated and finely controlled molecular procedures in which the PI3K/Akt/mTORC1 pathway activates the sterol regulatory element-binding protein (SREBPs) and changes the nutrition status of cancer cells. For instance, SREBPs are capable of regulating the synthesis of cholesterol and fatty acids and the expressions of key enzymes correlated with the synthesis. CD36, as a long-chain fatty acid receptor, can express exogenous fatty acid uptake, not just as an energy source, but also enhance intrusiveness in ESCC [31]. ACBP (Acetyl-CoA-binding protein) refers to the middle-long chain of the intracellular acetyl-CoA carrier protein that transports fatty acid in mitochondria for *β*-oxidation. ACBP has three types, including 1-acetyl-CoA-binding protein (1-ACBP), B-acetyl-CoA-binding protein (B-ACBP), and peripheral *β*-CoA-binding protein (PBR) [32]. It has been demonstrated that the three esophageal cancer genes increased significantly in the area of the malignant tissues of esophageal cancer. Furthermore, acetyl-CoA synthesis enzyme short-chain family member 2 (ACSS2) is capable of converting acetate into acetyl-CoA. The NRF2/ACSS2 axis will allow ESCC cells to exploit ethanol-derived acetate as their energy source. It can regulate downstream ethanol metabolism and metabolic reprogramming in ESCC, while inducing lipid synthesis and alcohol-related ESCC [23].

**Figure 1:**
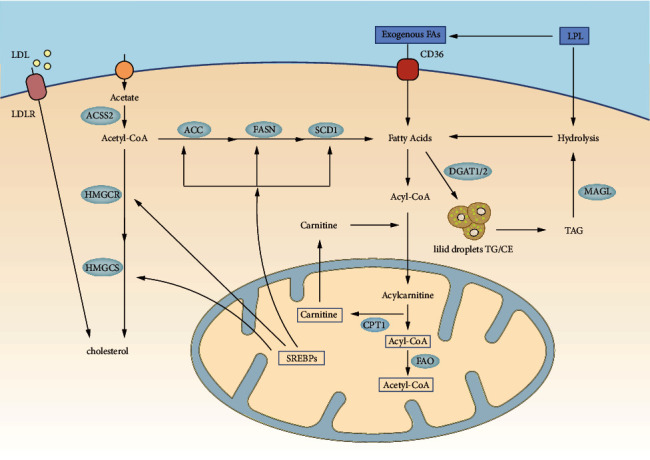
Overview of fatty acid metabolism in esophageal cancer cells. Lipid uptake can be achieved through multiple routes. ACLY, ATP citrate lyase; ACC, acetyl-CoA carboxylase; FASN, fatty acid synthase; SCD1, stearoyl-CoA desaturase 1; ACSS2, acetyl-CoA synthetase 2; ACS, acyl-CoA synthetases; CPT1, carnitine palmitoyl transferase 1; MAGL, monoacylglycerol lipase. TGs/CE, triglycerides/cholesteryl esters; TAG, triacylglycerol; FAs, fatty acids; FAO, fatty acid oxidation; LDLR, low-density lipoprotein receptor; HMG-CoA, hydroxy-3-methylglutaryl-CoA; HMGCR, hydroxy-3-methylglutaryl-CoA reductase; HMGCS, 3-hydroxy-3-methylglutaryl coenzyme A synthase; LDs, lipid droplets; SREBPs, sterol regulatory element-binding proteins.

As indicated by recent experiments, the lack of fructose-1,6-biophosphatase (FBP1) was correlated with the active metabolism of fatty acids in vitro and facilitated the proliferation, migration, and invasion of ESCC cells. In addition, protein expression levels for fatty acid synthase (FASN), acetyl-CoA carboxylase 1 (ACC1), and sterol regulatory element-binding protein 1C (SREBP1C) were correlated with triglycerides, neutral lipids, and fatty acid metabolism, while increasing significantly due to FBP1 regulation [33]. FASN is highly present in the phenotypes of the intestinal mucus of BE. Exposure to low pH and low bile acid in the esophagus may result in the expression of FASN in BE. SREBP1 can foster proliferation and induce epithelial-interstitial transition by activating stearoyl-CoA desaturase 1 (SCD1)-induced Wnt/*β*-catenin signaling pathways with carcinogenic effects on ESCC [34]. Cholesterol synthesized using mevalonic acid is critical to maintain the physical properties of the membrane, cell signaling, and physiological activity. The axis of the PI3K/Akt/mTORC1 signal can contribute to the acquisition of characteristics similar to those of cancer stem cells in squamous cell lines of the esophagus. Moreover, sterol regulatory element-binding protein 2 (SREBP2) raised HMG-CoA synthase, while MVA increased significantly. Cholesterol is catalyzed by acetyl coenzyme by 3-hydroxy-3-methylglutaryl (HMG)-CoA synthase and hydroxy-3-methylglutaryl-CoA reductase (HMGCR) to synthesize mevalonic acid. Esophageal cancer cells overexpressing HMGCR could facilitate cell growth and migration and colony formation [35]. The specific process is shown in Figure 1 and Tables1–3.

### 2.3. Lipid Metabolism-Related Enzymes and Regulatory Factor Targets

#### 2.3.1. ACSS2

Cancer cells are primarily dependent on acetate as a carbon source to synthesize fatty acids without oxygen-poor nutrients [98]. To be specific, acetic acid is catalyzed by ACSS2 to synthesize acetyl coenzyme A, which creates a key carbon source for cancer cells and overcomes oxidative stress and metabolism. ACSS2 refers to the only enzyme recycling acetate from desalination reactions to the cytoplasm and nucleus [36]. In a state of nutritional deficiency and oxidative stress, ESCC induces continuous growth and resistance to chemotherapy via the ACSS2/AMP-activated protein kinase (AMPK)/proliferating cell nuclear antigen (PCNA) pathways. Ming et al. demonstrated the function of nutritional stress (NS)-mediated acetyl coenzyme. ACSS2 can accelerate the recurrence of esophageal cancer patients and induce cisplatin resistance [37]. Moreover, it may serve as a potential biomarker to identify high-risk esophageal cancer patients. Ye et al. proved that differences in acetate levels in EC serum are correlated with choline levels in esophageal tissue, thus showing an improvement in lipid metabolism required for cell membrane synthesis in tumor cells [99]. Alcohol is also a critical element in the development of esophageal squamous cell carcinoma. As indicated by the experiment of Odera et al., NRF2/ACSS2 axis mediated the metabolic effect of alcohol drinking on ESCC [23]. In brief, ACSS2 targets in patients with esophageal cancer may create superior survival benefits. The above strategy may be required to be further validated in future clinical studies of inhibitors for esophageal cancer-targeting lipid metabolism with large sample sizes.

#### 2.3.2. ACC

Acetyl coenzyme refers to the second stage of fatty synthesis. It is an irreversible carboxylation to synthesize acetyl coenzyme A. ACC consists of two subtypes: ACC1 is locally cytoplasmic and synthesizes malonyl-CoA for the synthesis of fatty acids, while ACC2 is bound to the mitochondria's outer membrane, largely present in the lipid oxidation tissue. On the whole, acetyl coenzyme A is a variant inhibitor of carnitine acyltransferase 1 (CPT1) that regulates FAO [100]. Arising from their positions in subcells differ, the above two subtypes have distinct metabolic functions. ACC1 largely plays a certain role in lipid synthesis, while ACC2 primarily inhibits lipid degradation [101]. ACC1 locally generates malonyl-CoA, thus contributing to fatty acid synthesis. ACC2 binds to mitochondrial outer membranes and produces malonyl-CoA, and its action as an inhibitor for CPT1 regulates fatty acid *β*-oxidation [102]. In esophageal cancer, FBP1 acts as the major catalytic fructose-1,6-biphosphonate to produce a fructose-6-phosphate reversible reaction. Still, it is a surprising finding that the low expression of FBP1 can significantly promote fatty acid metabolism-related FASN, ACC1, and SREBP1C expression levels and eventually facilitate ESCC cell proliferation, migration, and invasion. Whether an effect on ESCC progression is exerted possibly by affecting fatty acid metabolism should be investigated in depth [4]. The experimental research of Zhao et al. demonstrated that ACC inhibitors as a potential anticancer drug have been increasingly recognized over the past few years. ACC's variant inhibitor, 5-(14-alkaloxygen)-2-furanic acid (TOFA), is cytotoxic to cancer cells and dose dependent to induce apoptosis. To the best of our knowledge, other ACC inhibitors (e.g., PF-05175157, Soraphen A, and CP-640186) have not been examined in esophageal cancer models [68, 69]. In brief, the development of additional novel sensitive ACC inhibitors as antitumor drugs is required.

#### 2.3.3. FASN

FASN is a crucial enzyme for the de novo synthesis of fatty acids, which plays an essential role in lipid metabolism and is correlated with tumor-related signaling pathways. In addition, it is capable of regulating the immune microenvironment, playing a certain role in epithelial-mesenchymal transition, and controlling tumor invasion and metastasis [38]. FASN expression in BE is significantly correlated with intestinal mucin phenotype, cell proliferation, accelerated angiogenesis, and cyclooxygenases-2 (COX-2) expression. FASN expression is likely to be a marker of an advanced risk of malevolence progression [39]. FASN upregulated in ESCC, and both knockdown and knockout of FASN significantly inhibited ESCC cell proliferation, which revealed a tumor exponent function for this gene in ESCC [40]. Downregulated FASN expression reduced the expansion and migration of esophageal cancer cells and should be considered a therapeutic strategy in esophageal cancer [41]. FASN may be an oncogeneic protein in esophageal cancer. For instance, FASN is expressed at remarkably increased levels in esophageal cancer, and C93 can inhibit the growth of EC [81]. C75, a first-generation synthetic small-molecule inhibitor of FASN, inhibits OSCC proliferation [39].

#### 2.3.4. SCDs

Stearoyl coenzyme A desaturated enzyme (SCDs) refers to an endoplasmic reticulum (ER) protein catalyzing the conversion of saturated fatty acids (SFAs) into ∆9-monounsaturated fatty acids (MUFAs) [103]. SFAs and MUFAs have been found as the main components of mammalian cell lipids, which consist of phospholipids (PLs), diacylglycerol (DAG), triglycerides (TAGs), and cholesterol esters (CE) (e.g., the essential components of biomembrane, sources of energy, and signaling molecules). Densely proliferating cancer cells have a high demand for MUFAs, mainly for the synthesis of new membrane PL, TAG, and CE. In esophageal cancer, lipid levels of MUFAs (primarily phosphatidylcholine) increased, while SFAs and PUFAs decreased. The accumulation of MUFAs observed in cancerous tissue overlaps with higher levels of SCD1 [104, 105]. MUFAs are capable of directly regulating CSCs via the Wnt/*β*-catenin pathway, which is one of the critical signaling pathways in esophageal cancer stem cells [106–108]. SREBP1 also promotes proliferation and induces epithelial-interstitial transition by activating the SCD1-induced Wnt/*β*-catenin signaling pathway, which has a carcinogenic effect in ESCC [34]. So far, several targeted SCD1 inhibitors, including BZ36, A939572, CAY-10566, and MF-438, have acted on a wide variety of tumors in tumor therapy. It is found that Oxaliplatin combined with A939572 can promote SCD1 overexpression and regulate hippo/YAP signaling pathways, which may more significantly inhibit tumor growth and metastasis in patients with cancer [109]. Accordingly, CSC-targeting treatment combined with SCD1 inhibitors may also act as a novel approach to treat esophageal cancer CSCs.

#### 2.3.5. SREBPs

The functions of SREBPs were confirmed as lipid synthetic transcription factors, specifically for synthesizing cholesterol and fatty acid [110]. SREBP1 turns out to be implicated in energy metabolism, including fatty acid metabolism, whereas SREBP2 is characterized by cholesterol synthesis. SREBP1 may equip the possibility for the diagnosis and therapy of ESCC. SREBP1 is required for generating ESCC cell expansion, migration, and aggression. Moreover, SREBP1 contributes to producing novel biomarkers and therapeutic targets for ESCC. SREBP1 exerts oncogenic effects in ESCC by stimulating proliferation and causing epithelial-mesenchymal transition through the SCD1-induced activation of the Wnt/*β*-catenin signaling pathway [34].

SREBP2, the head regulator for HMGCR, was upregulated in ESCC clinical samples. The overexpression of SREBP2 in ESCC cell lines boosted cancer cell growth, migration, and colony formation [42].

In esophageal squamous cancer, sterol regulatory element-binding transcription factor 1 (SREBF1), a critical regulatory protein for fatty acid metabolism, can regulate the synthesis of fatty acids, lipids, and glyphosate [111].

The above findings further indicated the fine regulation of esophageal squamous cancer, providing other potential therapeutic targets for ESCC. For instance, fatostatin, an active metabolite of tamoxifen, significantly repressed tumorigenesis via downregulating SREBP1 and epithelial-to-mesenchymal transition (EMT) markers [82].

#### 2.3.6. HMGCR

*(1) Mevalonate Pathways and Processes*. Cholesterol has been found as an essential element of lipid rafts, the primary venue for signaling regulation in cancer. Chelating membrane cholesterol is an efficacious anticancer strategy disrupting the functions of lipid rafts [43].

In the first step of identification, HMGCR, the speed-limiting enzyme of mevalonate pathway (MVP), is capable of converting HMG-CoA into mevalonate. In addition, MVP provides protein isoprenylation, which is critical to the downstream signal activity of small GTPases (Ras, Rho, or Rac) and to intracellular signal transduction [112]. Among MVP-targeted drugs, statins, as competitive inhibitors of HMGCR, regulate tumor cell growth and antitumor immune response. They can inhibit the proliferation of tumor cells and the migration and invasion of tumor cells. Regardless of the timing of statin use and the pathological subtype of esophageal cancer, statin was found to be correlated with a significant reduction in mortality in patients with esophageal cancer [113]. In addition, statins could serve as an adjuvant anticancer agent to treat esophageal cancer [114]. In ESCC cells, HMG-CoA reductase is critical to tumor genetics of esophageal cancer since the knockdown of HMG-CoA reductase expression can inhibit the growth and migration of EC [35], thus supporting the application of statins as anticancer agents for esophageal cancer. With the use of statins, extracellular signal-regulated kinase activation could be reduced, cell nuclear antigen could be proliferated, and D1 expression could be circulated to induce the apoptosis of ESCC [115]. Furthermore, statins could minimize cell survival and proliferation, manifest crucial metastasis markers, and induce the apoptosis of ECA cells [116, 117]. Besides, statins could increase radiation sensitivity and reverse the epithelial-interstitial transition of esophageal cancer cells, which confirmed the potential effect of overcoming radiation impedance in EC [118, 119]. Complementary statin therapy has also been reported to lead to better quality-adjusted life expectancy of ECA patients at lower costs. For the above reasons, it can be a promising cost-effective treatment [120]. It has been consistently demonstrated that statin is capable of improving the prognosis of esophageal cancer patients. Thus, statins can serve as promising complementary anticancer agents for patients with EC.

#### 2.3.7. CPT1

Carnitines play a significant function in carting long-chain fatty acids across the mitochondrial membranes and short-chain fatty acids across mitochondria into the cytosol and also take part in fatty acid oxidation (FAO) and energy metabolism [44]. The key rate-limiting enzyme of FAO, carnitine palmitoyltransferase I (CPTI), regulates FAO directly, thus stimulating cancer metabolic adaption. Determining the changes in carnitine and CPTI levels in patients with esophageal cancer may be considered as a novel screening method and a new treatment target for ESCC research results. Jin et al. discovered greatly enriched serum long-chain fatty acids in ESCC patients, thus confirming that the raised abundance of serum long-chain fatty acids might be the outcome of potent de novo fatty acid synthesis during expansion and metastasis of EC [45]. Shi et al. found a noticeable correlation between carnitine acyltransferase 1A (CPT1A) and poor prognosis in patients with ESCC, and CPT1A might be a predicting marker for ESCC [46]. Zhu et al. demonstrated that palmitoleic acid, palmitaldehyde, and isobutyl decanoate exhibited ideal diagnostic biomarkers [47]. Likewise, palmitoleic acid was indicated to exist in plasma metabolites of ESCC patients. In addition, *α*-methylacyl CoA racemase (AMACR) is also a long-chain fatty acid; its derivatives in FAO enzymes can be exploited to forecast neoplastic progression in BE [121].

### 2.4. Fatty Acids and Cholesterol Intake

Shyamsundar et al. reported that a chronic high-fat diet independently caused esophageal inflammation and metaplasia, the foremost steps in BE/EAC pathogenesis [119]. External fatty acids are carried into the esophageal cell via transportation means using specialized enzymes and proteins (CD36, low-density lipoprotein (LDL), and fatty acid-binding proteins 1 (FABP1)).

In general, cancer cells binding to membranes LDL-related receptors (LDLRs) via LDL are internalized and transported to advanced lysosomes. Ma et al. found that the LDL level is a negative predictive factor for ESCC. Further, LDL is an affordable and suitable biomarker that may help meet clinical requirements [70]. Then, lysosomal acid lipase (LAL) releases cholesterol to increase lipid metabolism. Low-density lipoprotein receptor-related protein 1B (LRP1B) will be removed in a wide range of tumors. New experimental results indicated that LRP1B most often occurs in esophageal cancer homologous deletion or hypermethylation transfer silencing via CpG island, thus resulting in the loss of function [122]. However, since LDL facilitates lymphatic metastasis, preoperative LDL serum levels are critical to predict ESCC results [70, 123]. In addition, low-density lipoprotein receptor-related protein 6 (LRP6) is considered a tumor promoter. The downregulation of LRP6 can inhibit cell migration, invasion, and EMT in ESCC cell lines [124].

CD36 refers to a cell marker initiating metastasis. Fatty acid receptor CD36 has been found as a marker and functional driver of lipid metabolic dependence metastasis [125]. ESCC cases show high levels of CD36 expression, which is significantly correlated with progression. If CD36 is artificially inhibited by intervention, the proliferation and invasiveness of ESCC cells will be significantly reduced. Since the viability of cells is dependent on the fatty acids produced under CD36 expression, ESCC cells with CD36 inhibition can only exploit specific essential amino acids (EAAs) as a source of energy. Thus, CD36 can be a promising biomarker and therapeutic target in ESCC. Furthermore, FAs may be a crucial regulator of energy dependence in ESCC cells [31]. However, existing research data are insufficient to demonstrate the clinical availability of CD36 expression, so in-depth research should be conducted to clarify the clinical use of CD36 receptor in ESCC patients.

Fatty acid-binding proteins (FABPs) are present in a wide variety of tissues and play a vital role in fatty acid metabolism. FABP1 is a rich acid-binding protein and a marker of intestinal and liver cells. Existing studies have reported that peroxisome proliferator-activated receptors (PPARs) are capable of regulating FABP1 and are indispensable in cell division, signal transduction, proliferation, and differentiation. FABP1 has different expression levels in dysplasia and EAC. This marker can act as critical diagnostic aids to determine the condition of cancer progression [48].

### 2.5. Lipid Droplet Formation and Lipolysis

Excess lipid in cells does not exist in unsterilized free fatty acids (FFAs) since high levels of FFAs have cytotoxic potential. Accordingly, cells store excess fatty acids and cholesterol in the form of neutral and inert biomolecules (e.g., sterol esters and TGs in cellular structures), which are termed lipid droplets. As reported by recent studies, LD abundance is correlated with increased invasive tumors and chemotherapy resistance to tumors [101]. As indicated by experimental studies, in patients with esophageal cancer with no stress, the constant increase in lipolysis and triglyceride cycle arises from the changes in nutrients brought by cancer, instead of the tumor [126]. In addition, fatty tissue around the tumor can have an effect on tumor development. Obese people are at higher risk of certain cancers (esophageal cancer). Cancer-associated adipocytes (CAAs) secrete adiponectin that can facilitate the adhesion, migration, and invasion of tumor cells [127]. Cancer cells and CAAs also undergo a dynamic exchange of metabolites. In particular, lipolysis is an essential mechanism for tumor cells to promote the release of fatty acids through CAAs. Triglycerides (TGs) act via three lipase types, including adipose TG lipase (ATGL), hormone-sensitive lipase (HSL), and monoacylglycerol lipase (MAGL), thus leading to the release of FFAs and glycerin [128].

Monoacylglycerol O-acyltransferase 2 (MGAT2) refers to a component of monoacylglycerol acyltransferase (MGAT) or acyl-CoA that catalyzes the synthesis of diacylglycerol, which is a prototype of triacylglycerol. Diacylglycerol O-acyltransferase 2(DGAT2) interacts with LDs, which may catalyze localized TG synthesis for LD growth. MGAT2 is downregulated at irradiations in well or poorly differentiated ESCC cells [129].

As demonstrated by recent investigations, the interaction of DGAT2 and MGAT2 can lead to the biosynthesis of lipid substrates for TGs [130]. According to Carrossini et al., LD increased with EAC evolution, which arose from the exposure of the esophageal epithelium to the hazard factors correlated with BE and EAC [49].

Fatty acids released by CAA are transferred to cancer cells and exploited in energy production via *β*-oxidation. The high availability of adipocyte cells in the tumor microenvironments supports tumor progression and uncontrolled growth [127]. In cancer cells, adipocyte cells act as significant sources of adiponectin and energy. Metabolic symbiosis mechanisms should be clarified, which can reveal novel therapeutic possibilities. LDs are the central organelle in s excess lipid stores energy through esterification. Whether this mechanism plays a role in esophageal cancer should be verified by in-depth research.

### 2.6. Lipid Raft

The lipid rafts in the cell membrane create a platform for mediating lipid signals to facilitate the transfer. Cancer cells are capable of combining lipid metabolism to support host cells in a microenvironment and facilitate distant metastasis [102]. They are highly dynamic and can serve as selective signal transduction to mediate lipid metabolism, cell survival, adhesion, metastasis, and tumor progression. The relative abundance of saturated fatty acids primarily accounts for the liquid order of lipid rafts, thus inhibiting the synthesis of lipid raft-related lipids. Cholesterol is a vital segment of lipid rafts, the prior media for signaling regulation in cancer, and chelating membrane cholesterol has been proven as an effective anticancer approach disrupting the roles of lipid rafts [131]. Lipid rafts are enriched in sphingolipids, but subtle changes in the raft lipidome are likely to appear to recruit specific proteins that eventually trigger specific signaling events [132]. Cancer cells contain higher levels of intracellular cholesterol and lipid rafts than their normal nontumorigenic counterparts. Numerous signal transduction processes are involved in cancer development. For instance, Gong et al. discovered that NF-*κ*B activation facilitated lipid raft construction in ESCC [133]. Lin et al. confirmed that repressing the construction of lipid raft-associatedRas-related C3 botulinum toxin substrate 1 (Rac1)/phosphatidylinositol 3-kinase (PI3K) p85*α*/Akt signaling complexes could inhibit stromal cell-derivedfactor-1*α* (SDF-1*α*)-induced aggression of EC [134]. Song et al. reported flotillin-1 stimulates tumor necrosis factor-*α* receptor signaling and activation of NF-*κ*B in ESCC [135].

### 2.7. Signaling Pathways Contributing to Lipid Metabolism Reprogramming

**Figure 2:**
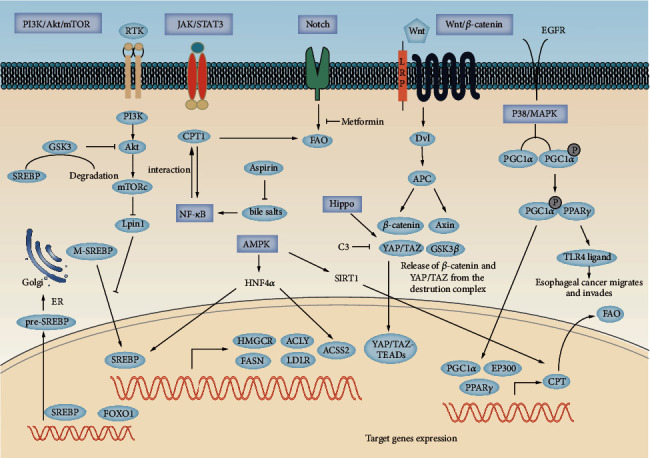
Targeting signaling pathways in EC. Schematic representation of the Wnt, Notch, AMPK, MAPK, and Hippo pathways in EC. Novel therapeutics (synthetic and natural) kill EC by targeting these signaling pathways or their components. GSK3*β*, glycogen synthase kinase 3*β*; DSH, Disheveled; APC, adenomatous polyposis coli; GGPP, geranylgeranyl pyrophosphate; YAP/TAZ, Yes-associated protein (YAP)/tafazzin (TAZ); JAK/STAT3, Janus kinase/signal transducers and activators of transcription 3; SIRT1, silencing information regulator 2-related enzyme 1 (sirtuin 1); HNF4A, hepatocyte nuclear factor 4 alpha; PGC1A, peroxisome proliferator-activated receptor gamma coactivator 1-alpha; PPAR-*γ*, peroxisome proliferator-activated receptor-*γ*; PI3K-Akt-mTOR pathway, the phosphatidylinositol 3-kinase (PI3K)-serine-threonine kinase (Akt)-mammalian target of rapamycin (mTOR) pathway; p38 MAPK, p38 mitogen-activated protein kinase; pre-SREBP, premature sterol regulatory element-binding protein; M-SREBP, mature sterol regulatory element-binding protein.

Pathways that maintain EC consist of Wnt/*β*-catenin, Notch, NF-*κ*B, and Hippo. The above pathways are involved in maintaining tissue homeostasis, and the regular renewal of stem cells and the deregulation of the above pathways leads to the formation of EC. Aberrant signaling of the critical pathways was correlated with metabolic reprogramming of lipids in esophageal carcinoma. The specific process is shown in Figure 2).

#### 2.7.1. NF-*κ*B Signaling Pathway

The incidence of esophageal adenocarcinoma tends to increase during esophageal reflux inflammation, metaplasia, and dysplasia. Nuclear factor-kappa B (NF-*κ*B) is critical to inflammation and tumorigenesis. As reported by existing studies, the NF-*κ*B signaling pathway on the lipid raft of esophageal cancer cells plays a crucial role, and abnormal lipid metabolism changes can regulate the lipid raft's function. The above studies revealed that NF-*κ*B is of critical importance to lipid metabolism. On the one hand, N-3 polyunsaturated fatty acids can precisely change the composition of membrane lipid rafts. The enrichment of N-3 PUFA changes the transverse organization of membrane signaling components (lipid rafts), thereby affecting downstream signaling, T-cell activation, transcriptional activation, and cytokine secretion [136].

On the other hand, cholesterol acts as a crucial lipid component for the integrity and function of the raft, through which it locates receptors and promotes cell signaling [137]. Moreover, exogenous high-fat dietary intake also induces early BE in mice through lipid group remodeling [138]. The above theories all show that changes in cholesterol and fatty acids in cancer cells can affect a wide range of functions of lipid rafts. In addition, lipid rafts play an essential role in promoting the development of esophageal squamous cell carcinoma in NF-*κ*B signaling. Fang et al. reported that the Janus kinase/signal transducer and activator of transcription 3 (JAK/STAT3) pathway is a vital pathway connecting cancer growth through the NF-*κ*B p65 subunit and COX-2 [139]. The STAT3 pathway could serve as a novel target for ESCC cancer treatment and prevention. As revealed by the latest experiments, aspirin (a nonsteroidal anti-inflammatory drug) blocked the increase in acid- and bile salt-induced NF-*κ*B activation, p65 nuclear translocation of patients with esophageal squamous cells of BE [140].

#### 2.7.2. PI3K/Akt/TOR Signaling Pathway

The PI3K/Akt signaling pathway facilitates cell growth through the activation of mTORC1. Positive expression of mTOR is significantly correlated with the depth of tumor infiltration, staging of TNM, degree of differentiation, and metastasis of lymph nodes in squamous cell carcinoma of the esophagus. Furthermore, the PI3K/Akt/TOR pathway is critical to lipid metabolism reprogramming in esophageal cancer. The PI3K/Akt/TOR pathway regulates the biosynthesis of fatty acids and cholesterol via SREBP1 and promotes key nascent lipids enzymes (e.g., ACC, FASN, and SCD) to varying degrees [103, 134]. Moreover, SREBP1 is correlated with increased tumorigenesis and poor prognosis of esophageal cancer cells [106]. According to Zhong et al., SREBP2 could promote ESCC cell growth, migration, and colony formation by regulating HMGCR and the synthesis of cholesterol. In addition, PI3K/Akt upregulates the expression of Forkhead Box O1 (FOXO1), thus leading to the beginning of SREBP transcription [135]. Turk et al., lysophosphatidylcholine acyltransferase 1 (LPCAT1) could regulate the expression of cholesterol synthase squalene monooxygenase (SQLE) by facilitating the activation of PI3K, promoting the entry of SREBP1 into the nucleus, and eventually achieving re-cholesterol metabolism reprogramming, thus promoting the occurrence and development of esophageal squamous cell carcinoma [136].

#### 2.7.3. Wnt/*β*-Catenin Signaling Pathway

In the normal physiological state, the Wnt/*β*-catenin signaling pathway can regulate downstream genes involved in essential cell biological functions (e.g., cell proliferation, differentiation, apoptosis, and cell death) [141]. However, numerous findings suggested that abnormal *β*-catenin expression may be poor underlying factors affecting esophageal cancer's ability to progress, metastasize, and invade [142].

Recent studies have reported the interactions between the Wnt/*β*-catenin pathway and abnormal lipid metabolism reprogramming, which include proliferation-activated receptor *α* (PPAR*α*) that plays a certain role in fatty acid transport and mitochondrial *β*-oxidation [139]. As reported by Senni et al., *β*-catenin could determine the energy source cells that are specifically used to support growth by inhibiting the regulation of PPAR*α* [140]. According to the experiment of Xu et al., PPARg could regulate FAO by negatively regulating CPT1A, that is, an essential enzyme for FAO, thus having a further effect on the polarization of anti-inflammatory (M2) macrophages [141]. Moreover, abnormal lipid metabolism reprogramming is capable of activating signaling pathways to promote the occurrence and development of esophageal cancer. As indicated by Wang et al., SREBP1/SCD1 could regulate proliferation, invasion, and metastasis via the Wnt/*β*-catenin signaling pathway in ESCC [27].

#### 2.7.4. AMPK Signaling Pathway

AMPK refers to a central regulator of cellular metabolism and energy homeostasis in mammalian tissues [142]. When cellular energy levels decrease, AMPK is activated; then by activating critical enzymes involved in fatty acid metabolism (ACC1 and SREBP1c), it further exerts its early anti-syntheticpro-breakdown effect, thus leading to an increase in ATP levels. In addition, hepatocyte nuclear factor 4 alpha (HNF4*α*) is a crucial regulator of the oxidation of mitochondrial FAO, cholesterol, and lipoprotein metabolism [143]. Barrett's metaplasia is the only known morphological precursor to esophageal adenocarcinoma, replacing the stratified squamous epithelium with columnar epithelium. Colleypriest et al. found that AMPK could activate HNF4*α*, thus facilitating a critical early step in Barrett's metaplasia [144]. According to the study by Mi et al., in the case of nutritional stress when the AMPK pathway was activated, the nutrient supply and chemotherapy tolerance of ESCC cells were significantly upregulated. The above phenomenon largely arose from the activation of ACSS2 and PCNA, an essential DNA replication and repair regulator [31]. Sirtuin 1 (SIRT1) is an NAD+-dependent histone deacetylase that plays an essential role in regulating lipid metabolism and can work synergistically with AMPK to regulate lipid metabolism. When cells experience nutritional stress, SIRT1 can produce an energy supply by promoting FAO, thus probably leading to changes in lipid metabolism on a wide variety of pathophysiological backgrounds [145]. The study by Ma et al. found that the expression level of the SIRT1 protein was significantly correlated with TNM staging and lymph node status in ESCC patients [146].

#### 2.7.5. JAK/STAT3 Signaling Pathway

As indicated by recent reports, STAT3-mediated lipid metabolism takes on a critical importance to cancer progression. According to Yan et al., the effects arising from PUFAs on myeloid-derived suppressor cells (MDSCs) were dependent on STAT3 signaling, and blockage of STAT3 phosphorylation nearly reversed the effect of PUFAs on MDSCs [143]. Li et al. concluded that the effects arising from PUFAs on MDSCs were dependent on STAT3 signaling, and that the blocking of STAT3 phosphorylation nearly reversed the effect of PUFAs on MDSCs. Moreover, COX-2, also correlated with JAK/STAT signaling, is involved in tumorigenesis [144]. Fang et al. identified the JAK/STAT3 pathway as an essential pathway linking cancer growth across the NF-*κ*B p65 and COX-2 subunits. The STAT3 pathway could be a novel target for cancer treatment and prevention in ESCC [139].

Zhao et al. reported that nimesulide downregulated COX-2 and survived expression and improved caspase-3 expression in ESCC by activating JAK2/STAT3 channel [147]. Ishimura et al. found that FASN was expressed in the intestinal mucin phenotype of BE, in which Barrett's glandular cells had a high COX-2 expression [99].

#### 2.7.6. Hippo Signaling Pathway

The Hippo-YAP pathway regulates mammals' organ size, tissue homeostasis, as well as tumorigenesis [145]. The transcriptional coactivators Yes-associated protein 1/transcriptional coactivator with PDZ-binding motif (YAP/TAZ) is a downstream transcriptional coactivator of the Hippo signaling pathway. YAP/TAZ interacts with mature SREBPs in the nucleus, thus enhancing the transcriptional activity and upregulating the expressions of crucial fatty acids and cholesterol enzymes (e.g., HMGCR and FASN) [146]. When statin inhibits the activity of HMGCR in the mevalonate pathway, the transcriptional activity of YAP/TAZ is inhibited [148]. Moreover, SCD1 promotes the synthesis of unsaturated fatty acids, thereby further activating the Wnt ligand and ultimately promoting the accumulation of *β*-catenin and YAP/TAZ in the nucleus, playing a role in transcriptional regulation [149]. In esophageal cancer, the YAP1 protein is generally overexpressed and positively correlated with the histological grading, staging, diameter, and overall and disease-free survival of ESCC; the downregulation of YAP1 will promote the apoptosis of ESCC cells or inhibit their proliferation and invasion [147].

### 2.8. The Link between Lipid Metabolism and Immune Response in the Esophageal Cancer Microenvironment

The esophageal mucosa, an organ directly exposed to the risk of foodborne infection, has a unique group of innate immune cells that are critical to maintain esophageal stability and carry out immune defense. ESCC is enriched in immune-suppressive cell populations (e.g., Tregs; exhausted CD8+ T cell, CD4+ T cell, and NK cells; M2 macrophages; and DC cells) [150]. CD8+ T-cell infiltration has been generally part of the host's antitumor immune response, whereas it can facilitate the exacerbation of chronic inflammation and the development of tumors. CD8+ T cells in EAC patients may indicate poor inflammatory responses in the surrounding tissue microenvironment, which can be conducive to the patients' prognoses [151]. CD8+ T cells lacking SREBP activity are expected to have insufficient cholesterol. Phospholipids and cholesterol, the raw materials in cell membranes, are insufficient to generate sufficient antigen stimulation, thus destroying the tumor-killing function of infiltrating CD8+ cytotoxic T cells. Regulatory T cells are capable of inhibiting anticancer immunity, limiting the protective immune monitoring of tumors, and facilitating the occurrence and development of tumors. Foxp3+ T cells can uptake more long-chain fatty acids (Tconv) than traditional T cells, which protect themselves from the potential damage of acylated FFAs. Foxp3 and Toll-like receptor signaling can balance Treg cell anabolic metabolism for suppression [152].

As proven by the experiments by Liu et al., Treg cells improved the SREBP1-dependent metabolic fitness of tumor-promoting macrophages by repressing CD8+ T cell-derivedinterferon-*γ* [153].

M1 macrophages could induce FA synthesis, while anti-inflammatory signals favoring M2 macrophages would facilitate FAO [154, 155]. M2-like TAMs have been suggested to inhibit antitumor immunity, induce angiogenesis, and facilitate cell migration [156]. An increased proportion of TAMs in ESCA in esophageal cancer is correlated with high aggressiveness and poor prognosis of patients [157].

**Figure 3:**
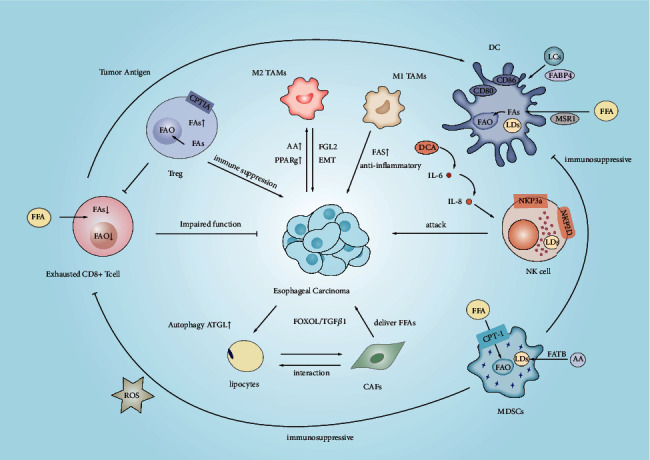
Lipid metabolism reprogramming in the esophageal cancer microenvironment affects the anti‐/pro‐tumoral functions of immune cells. MDSC, marrow-derived suppressor cells; DCs, dendritic cells; FAO, fatty acid oxidation; FAS, fatty acid synthesis; TAMs, tumor-associated macrophages; FFA, free fatty acid; Tregs, regulatory T cells; NK Natural killer cells; AMPK, AMP-activated protein kinase; IL-10, interleukin 10; TGF-*β*, transforming growth factor-*β*; STAT3, transcription 3; LPL, lipoprotein lipase; NF-*κ*B, nuclear factor; FABPs, fatty acid-binding proteins; LRP4, lipoprotein receptor-related protein 4; PPAR, peroxisome proliferator-activated receptors.

As revealed by Zhou et al., monocyte chemotactic protein 2 (MCP2) secreted by M2 macrophages could activate the NF-*κ*B signaling pathway, facilitate ESCC cell migration and invasion, and induce the EMT process [158]. The specific process is shown in Figure 3.

### 2.9. Esophageal Cancer Lipid Metabolism on T Cells in the Immune Microenvironment

Different subsets of T cells consist of effector cells (Teff), regulatory cells (Treg), and memory cells (Tmen). Different metabolic characteristics have an effect on the differentiation and function of T cells. Existing studies have shown that Teff cells rely on FAS. In contrast, Foxp3+ Treg cells and CD8+ Tmem cells depend on the production of long-chain fatty acids (LCFAs). In EC, the B7/CTLA-4 signaling pathway activates the tumor or matrix derived from chemokines (e.g., CCL20 (C-C motif chemokine ligand 20)) [159], activating and recruiting Treg. Subsequently, Treg is capable of secreting immunosuppressive factors interleukin-10 (IL-10), transforming growth factor beta (TGF-*β*) [160], etc. Moreover, lipid accumulation in immune cells can facilitate oxidative metabolism. For instance, lipid accumulation in dendritic cells reduces their ability to process and present antigens, thus reducing the ability to stimulate T cells, which reveals that lipid accumulation in the tumor microenvironments can have an effect on the function of immune cells. In addition, high esterification rates of cholesterol in tumors can reduce the response of T cells. Elevated cholesterol levels of CD8+ T-cell membranes are likely to facilitate their proliferation and improve their effect function, which is consistent with the role played by FAO as indicated by our consideration. As reported by the experiments by Pearce et al., mice lacking TNF receptor-associated factor 6 (TRAF6) could not raise FAO, and the production of Tmem also had severe defects. AMPK could reverse the above situation, since it promoted the induction of FAO in CD8+ T cells, while playing the most critical role in facilitating the production of Tmen. AMPK is capable of regulating two pathways (i.e., FAS and FA). To be specific, it is capable of inhibiting FAS by reducing the formation of malonyl-CoA of ACC1, while it can hinder ACC2 (correlated with mitochondria), a rate-limiting enzyme in FAO. It has been confirmed that there is a direct correlation between AMPK activation and the upgrading of FAO via the classic ACC2-CPT1 axis in liver cells. Whether there is a related lipid metabolism pathway in esophageal cancer should be explored in depth. Besides, AMPK can indirectly promote FAO by upregulating the expression of CPT1 in a peroxisome proliferator-activated receptors (PPAR)-peroxisome proliferator-activatedreceptor-coactivator (PGC)-1-dependent mechanism. A wide range of fatty acids have been reported to facilitate the differentiation and proliferation of T cells in the intestine. Medium and long-chain fatty acids are capable of supporting the differentiation of pro-inflammatory Th1 and Th17 cells, while short-chain fatty acids facilitate the development of Treg cells. The divergence of metabolic reprogramming is of high importance to effectively label the fate of different T cells. Furthermore, strengthening FAO by improving AMPK activity or inhibiting rapamycin's mammalian targets will increase the number of memory T cells. Whether the functional results of the programmed cell death protein-1(PD-1) connection are correlated with T-cell reprogramming to specific metabolic pathways remains unclear. In our previous study, we have investigated the metabolism of T cells receiving PD-1 signals and found that they did not play a certain role in the metabolism of glycolytic enzymes, glutamine hydrolysis, or branched-chain amino acids, but showed higher FAO rates. Moreover, FAO maintains the survival and persistence of PD-1 signaling T cells in patients with chronic infection and cancer and reverses PD-1 blocking and revitalizes their abilities [161]. Our results revealed the accumulation of cholesterol, phospholipids, and saturated phospholipids in the T-cell receptor activation domain, which confirmed that lipid components in the immune cell membrane are involved in their function and signal transduction. CD8+ T cells could facilitate inflammation and tumor development in esophageal cancer and reduce tumor growth via cancer monitoring. The exact mechanism of cytotoxic CD8+ T cells impacted by gastroesophageal reflux disease may be complex. According to existing EAC studies, the abundance of CD8+ tumor-infiltrating lymphocytes (TILs) is correlated with prolonged survival, better pathological responses to neoadjuvant chemotherapy, lower lymph node metastasis rates, and better prognosis. In general, cytotoxic CD8+ T cells could release interferon, granzyme A, granzyme B, and Fas ligand to induce the apoptosis of the target cells [162]. Collectively, directly targeting cellular FA metabolism may serve as a promising strategy to control T-cell response in vivo. However, in subsequent studies, the role of FAS and FAO in T-cell subsets and other immune cell types should be further analyzed, and the effects arising from FA metabolism in different stages of inflammatory response should be clarified.

### 2.10. Esophageal Cancer Lipid Metabolism on Tumor-Associated Macrophages (TAMs) in the Immune Microenvironment

Macrophages are an important part of the tumor immune system. Tumor-associated macrophages can be enlisted and cultivated to exert antitumor or proto-tumor functions during tumor initiation and progression [163]. Esophageal cancer is not an exception to this rule. Inflammatory (M1) macrophages are capable of sustaining inflammatory responses and killing pathogens, relying primarily on aerobic glycolysis and fatty acid biosynthesis. Using predominantly aerobic glycolysis and fatty acid production, inflammatory (M1) macrophages may maintain inflammatory responses and destroy pathogens. Experimental studies by Jiang et al. found that M1 macrophages can be utilized to predict a patient's prognosis for ESCC by preventing ESCC cell migration and invasion [164]. By integrating transcriptomic and lipid metabolomic profiling, a large number of fatty acids and lipid mediators are pan-agonists of peroxisome proliferator-activated receptors (PPARs), and arachidonic acid, unsaturated fatty acid, significantly affects esophageal squamous cell carcinoma by regulating peroxisome proliferator-activated receptor gamma (PPARg) and OXPHOS M2 polarization in macrophages [165].

Anti-inflammatory (M2) macrophages, in contrast, mediate tissue repair and inflammation lysis by switching their metabolism to FAO and oxidative phosphorylation [166]. Yuan et al. found that M2-liketumor-associated macrophages could generate an immunosuppressive tumor microenvironment by secreting fibrinogen-like protein 2 (FGL2) to induce the occurrence and progression of esophageal cancer [157]. In addition, M2 macrophages might encourage ESCC cell invasion and migration, leading to epithelial-mesenchymal transition (EMT) and a poor prognosis for ESCC patients [167, 168]. Thus, the specific mechanism by which lipid metabolism enables TAMs to actively or passively change their phenotype to adapt to the microenvironment is poorly understood, but this property could provide not only survival opportunities for tumor cells but also facilitate tumor therapy.

### 2.11. Esophageal Cancer Lipid Metabolism on Dendritic Cells (DC) in the Immune Microenvironment

The DCs distributed in the esophageal mucosa are generally Langerhans cells (LCs), which mature rapidly into specialized atrial premature complexes (APCs) after capturing tumor-associated antigens, thus leading to T-cell activation and immune response [169]. DC immune function in EC patients is impaired both at the circulation and tumor sites, accompanied by decreased CD80 and CD86 expression [170]. The above infiltrations of a decrease in DC activity and function are correlated with the overexpression of the mutated p53 protein in tumors [171]. The study by Brix S et al. demonstrated that differences in dietary fatty acids affect the overall level of CD4(+) T-cell activation induced by dendritic cells. Moreover, DC stimulation can also trigger changes in CD80 and CD86 levels [172]. There was a significant increase in DC density in adenocarcinoma compared to benign BE, thus revealing the potential of DC in EAC dysplasia and tumorigenesis [173].

Among the tolerated DCs, an increase in OXPHOS and FAO is correlated with IL-6. The experiments of Zhao et al. demonstrated that fibroblasts promote the production of monocytic-myeloid-derived suppressor cells (M-MDSCs) by activating the STAT3 signal secretion of IL-6 and emphasized that fibroblasts work together with M-MDSCs to promote the production of cisplatin resistance, thus providing a potential opportunity for inhibiting STAT3 signal reversal resistance in ESCC [174]. Tumor-derived FAs are capable of increasing OXPHOS and FAO through CPT1A transport or activation of PPAR*α*. The activation of PPAR*α* can also facilitate the formation of LD, which has a higher oxidation content of PUFAs. According to the experiments of Wang et al., the expression levels of PPAR-*α* in BE and esophageal adenocarcinoma samples were 5.9130 and 2.0314 times higher than those of normal esophageal squamous epithelial cells, respectively. The upregulated expression of PPAR-*γ* may be critical to develop BE and ECA from normal cells [175].

Exogenous FAs are transported through macrophage receptor 1 (MSR1) and fatty acid-bindingprotein-4 (FABP4), supplementing the above LDs and increasing their oxidized PUFA content. The above LDs and tumor-derived FAs themselves impair cross-presentation by inhibiting MHCI surface expression. Inflammatory responses occur at the early stages of EAC and even in BE. DCs significantly impact the immune response of esophageal adenocarcinoma [176]. Changes in fatty acid metabolism affect the “switch” function of DC, whether immune activation or immunosuppression is selected. Moreover, wingless-type protein 5a (Wnt5a) can add FAO and OXPHOS by downregulating PPAR*γ* [177]. As indicated by the experimental studies of Lyros et al., abnormal regulation of the Wnt5a/receptor tyrosine kinase-like orphan receptor 2 (ROR2) signaling pathway could facilitate the development of Barrett-associated esophageal adenocarcinoma [178]. As reported by Wu et al., Wnt5a can improve the invasive ability of ESCC cells via a wide variety of signaling pathways [179].

### 2.12. Esophageal Cancer Lipid Metabolism on Myeloid-Derived Suppressor Cells (MDSCs) in the Immune Microenvironment

Tumor cells can facilitate fatty acid uptake and synthesis in the TME, recruiting fat cells to produce excess exogenous fatty acids, thus creating a lipid-rich environment for the infiltration of MDSCs. MDSCs then adopt FAO as their primary energy. Lipid-rich TME can expedite the development of MDSCs and enhance their immunosuppressive function. As reported by existing studies, tumor-derived cytokines (granulocyte macrophage colony-stimulating factor (GM-CSF) and granulocyte colony-stimulating factor (G-CSF)) subsequently can induce the expression of lipid transporters via the STAT3 and STAT5 signaling pathways, thus increasing the uptake of high concentrations of lipids in the tumor microenvironment. Intracellular lipid accumulation increases oxidative metabolism and activates immunosuppressive mechanisms. Tumor-associated MDSCs upregulate FAO as the primary energy source. Moreover, CD36 uptakes fatty acids and supports the expression of FAO key enzymes (CPT-I). Lipid metabolism is correlated with the inhibitory function of MDSCs. Fatty acid uptake, fatty acid *β*-oxidation (FAO), fatty acid synthesis, and lipogenesis increased MDSCs, leading to an upregulation of immunosuppressive function [180]. Inhibition of the STAT3 or STAT5 signaling pathway or deletion of the fatty acid transpose CD36 gene inhibits the activation of oxidative metabolism and induction of immunosuppressive function in tumor infiltration MDSCs and leads to delays in tumor growth dependent on CD8+ T cells [181]. Liu et al. experimentally demonstrated that upregulation of phosphorylated STAT3 (p-STAT) and phosphorylated STAT5 (p-STAT) expression might be correlated with esophageal squamous cell carcinoma development [182]. In patients with ESCC, the number of circulating MDSCs is elevated, accompanied by high expression of programmed death-ligand 1(PD-L1). MDSCs exert immunosuppressive effects on T cells through the PD-1/PD-L1 pathway and are correlated with tumor burden, lymph node metastasis, and tumor staging [183]. Adeshakin et al. demonstrated that fatty acid transporter 2 (FATP2) could endow PMN-MDSCs with the function in cancer by upregulating arachidonic acid metabolism. FATP2 plays a crucial role in regulating lipid accumulation-induced oxygen species in MDSCs, and targeting FATP2 in MDSCs can serve as a novel therapeutic method to enhance anti-PD-L1 cancer immunotherapy [184].

### 2.13. Esophageal Cancer Lipid Metabolism on NK Cell in the Immune Microenvironment

NK cells refer to the first responders to innate immune responses. Pro-inflammatory cytokines secreted in the tumor immune microenvironment can activate NK cells and recruit more immune cells [185]. Studies have shown that NK cell infiltration density in EC TME positively correlated with overall survival (OS) and good postoperative prognosis [186, 187]. NK cells in EC TME are capable of recognizing and killing tumor cells via the NKp30/B7-H6 pathway, thus primarily facilitating NK cell-mediated immune responses [186]. However, lipid metabolism is critical to the immune microenvironment of esophageal cancer. As indicated by Lim Kee et al., the deoxycholic acid (DCA) also stimulated the production of interleukin-6 (IL-6) and interleukin-8 (IL-8) in esophageal adenocarcinoma cells via raft-related signaling, thus facilitating esophageal metaplasia and dysplasia [188]. As proven by the experiments by Wu et al., the activation of STAT3 signaling by IL-6 and IL-8 secretion led to the downregulation of receptors (NK protein 30 (NKp30) and NK group 2D (NKG2D) receptors) on the surface of activated NK cells, thus leading to impaired NK cell function and ultimately resulting in the development of esophageal squamous cell carcinoma [189].

After NK cells were activated, the mTORC1 signaling pathway was modulated to produce interferon-gamma (IFN*γ*) and granzyme B, which performed their effect function [190]. Their activation was consistent with increased ATP citrate lyase (ACLY) expression and citric acid transport to cytoplasm, correlated with acetylation and epigenetic control [191]. According to the experimental studies of Carrossini et al., in BE and EAC, associated inflammatory stimulation would lead to a gradual increase in LD and a significant increase in IL-8 secretion. This phenomenon reveals an interaction between lipid droplets and inflammatory mediators, thus facilitating BE and EAC's occurrence and development [49]. Moreover, exogenous lipids could disrupt this metabolic program and have a negative effect on their effector function and ability to respond to stimuli, especially in the case of obesity [192–194].

### 2.14. Esophageal Cancer Lipid Metabolism on Cancer-Associated Fibroblasts (CAFs) in the Immune Microenvironment

CAFs have been more commonly induced in EMT [195]. CAFs secrete immunosuppressive and pro-angiogenesis factors in TME [68, 69], which has been found in recent literature and thus indicated that they may play a role in lipid breakdown and chemotherapy resistance in esophageal cancer. As demonstrated by the experiments of Qiao et al., IL-6, primarily from cancer-associated fibroblasts, upregulated chemokine receptor type 7 (CXCR7) expression through activators of the STAT3/NF-KB pathway, which could play a critical role in chemical resistance [196]. As reported by a previous study, fat cells, once activated by cancer cells, will eventually secrete higher levels of pro-inflammatory cytokines (e.g., IL-6) [197, 198]. Cancer cells could also secrete the above pro-inflammatory cytokines and help induce the release of fatty acids stored in fat cell triglycerides since they are considered strong lipolytic factors [199, 200]. The upregulation of IL-6 can amplify the metabolic cross-disorder STAT3 pathway between the two cells through the IL-6 signal, and CD36 has recently been shown to be a downstream target for activating STAT3, which will further promote fatty acid uptake of cancer cells [201, 202]. If so, metabolically activated adipose tissue macrophages also secreting IL-6 at high levels may play a role in this axis [203]. As demonstrated by Zhao et al., cancer-associated fibroblasts induce bone marrow-derived macrophages via the IL-6/STAT3 pathway to facilitate drug resistance in cisplatin of esophageal squamous cell carcinoma [174]. Zhang et al. confirmed the chemical tolerance of cancer-associated fibroblasts through FOXO1/transforming growth factor (TGF-*β*1) signaling ring-mediated esophageal squamous cell carcinoma [204]. A FOXO1 transcription factor is found to play a vital part in cellular stress response and is correlated with plentiful nutrient regulation processes. FOXO1 regulates lipid metabolism in adipose tissue by regulating fat cell size and the expression of AT-specific genes, including ATGL, which is a rate-limiting enzyme playing a role in the breakdown and storage of TG stored into LDs [205]. FOXO1 is critical to fatty acid-induced oxidative stress in fat cells [206].

## 3. Conclusion

In this review, we elucidate how abnormal lipid metabolism has an effect on the occurrence and development of esophageal cancer. This review is original and innovative and will present more insights into the pathogenesis of esophageal cancer. Hopefully, this review can lay a theoretical basis for improving ESCC prognosis models, clarifying drug resistance mechanisms, and formulating corresponding targeted therapies.

## Figures and Tables

**Table 1:** Cancer cell biomarkers for prognosis of esophageal cancer.

Markers	Cancer Type EAC/ESCC	Significance	Name	Marker for diagnosis or prognosis	Reference
ACSS2	ESCC	ACSS2 can recycle acetate (including both protein and metabolite deacetylation reactions) to produce acetyl-CoA, which is a raw material for fatty acid and cholesterol synthesis [36].	Lei et al.	The expression of ACSS2 is closely related to the prognosis of patients with ESCC.	[37]

ACC	ESCC	Acetyl-CoA carboxylase catalyzes the conversion of acetyl-CoA to malonyl-CoA. ACC promotes FA synthesis and the energy metabolism.	Zhao et al.	Downregulation the expression of p-ACC is associated with tumor cell differentiation in ESCC.	[50]

FASN	ESCC	FASN is a key enzyme for lipid metabolism and is associated with tumor invasion and metastasis [38].	Ishimura et al.	FASN expression was associated with the risk of malignancy progression.	[39]
	Barrett's esophagus/OAC		Wang et al.	FASN promotes the development of esophageal squamous cell carcinoma.	[40]
	ESCC/OAC		Zhou et al.	FAS has the potential to be oncogenic in EC.	[41]

SCD1	ESCC	SCD1 appears to be a significant player in the development of tumor and may be a promising target for anticancer therapy [51].	Zemanova et al.	Compared with healthy patients, both saturated and monounsaturated fatty acids were increased in esophageal cancer patients due to increased activity of SCD1	[52]

ELOVL5		ELOVL5 is a key enzyme for de novo synthesis of long-chain unsaturated fatty acids.	Zhao et al.	ELOVL5 is upregulated in EC and is associated with a poor prognosis in patients.	[53]

CD36	ESCC	CD36-driven lipid metabolic reprogramming and tumor development [54].	Yoshida et al.	CD36 was associated with tumor invasion and metastasis in ESCC.	[31]

			Wang et al.	Overexpression of SREBP1 was significantly correlated with tumor differentiation and lymph node metastasis of ESCC.	[34]

SREBP1	ESCC	SREBP1 may provide the potential for the diagnosis and treatment of ESCC.	Shao et al.	SREBP1 can be used as an independent prognostic marker for ESCC.	[55]

		SREBP1 exerts oncogenic effects in ESCC by promoting proliferation and inducing epithelial-mesenchymal transition via the SCD1-induced activation of the Wnt/*β*-catenin signaling pathway.	Wang et al.	SREBP1 contributes to the development of novel biomarkers and therapeutic targets for ESCC.	[34]

SREBP2	ESCC	Sterol regulatory element-binding protein 2 (SREBP2), the master regulator for HMGCR, is upregulated in ESCC clinical samples.	Zhong et al.	SREBP2 is closely related to ESCC tumorigenesis.	[42]

CPT1A	ESCC	CPT1A acts as a key enzyme in fatty acid oxidation and regulates tumor energy metabolism.	Shi et al.	CPT1A is capable of potential biomarkers for the risk prediction for ESCC.	[46]

Carnitine		Levels of octanoylcarnitine, lysoPC (16 : 1), and decanoylcarnitine are closely related to the effectiveness of ESCC treatment [44, 56].	Li et al.	Carnitine is capable of potential biomarker for the risk prediction and early detection of ESCC.	[57]
l-Carnitine/acylcarnitin	ESCC		Xu et al.	Acylcarnitine is a potential biomarker of ESCC	[58]

Octanoylcarnitine, lysoPC (16 : 1), and decanoylcarnitine	ESCC	Levels of octanoylcarnitine, lysoPC (16 : 1), and decanoylcarnitine have been reported to be associated with the treatment effects and are identified as potential biomarkers.	Xu et al.	Levels of octanoylcarnitine, lysoPC (16 : 1), and decanoylcarnitine have been reported to be associated with the treatment effects and are identified as potential biomarkers.	[59]

LDs	Barrett's esophagus/OAC	Lipid droplets are dynamically active and control lipid homeostasis.	Carrossini et al.	LDs are increased along EAC evolution as a consequence of the exposure of the esophageal epithelium to the risk factors associated with BE and EAC.	[49]

Cholesterol	ESCC/OAC	Cholesterol is a useful component of lipid rafts and controls various signaling pathway.	Zhu et al.	Cholesterol can be used as a potential biomarker of EC.	[43]

LPCAT1	ESCC	LPCAT1 regulates SREBP1 and SREBP2-related signaling pathways in ESCC cells.	Tao et al.	LPCAT1 may be a useful biomarker for ESCC diagnosis and prognosis.	[60]

HMGCR	ESCC	3-hydroxy-3-methylglutaryl coenzyme A reductase (HMGCR) is the rate-limiting enzyme in cholesterol biosynthesis.	Zhong et al.	HMGCR may be an important therapeutic target for esophageal squamous cell carcinoma.	[35]

FABP1	OAC	Fatty acid-binding proteins (FABPs) are intracellular proteins that bind long-chain fatty acids (FA) and are related to immunometabolic diseases.	Srivastava et al.	FABP1 can clearly discriminate Barrett's esophagus from columnar lined esophagus.	[48]

HNRNPA2B1	ESCC/EAC (both)	HNRNPA2B1 upregulates ACLY and ACC1 and promotes ESCA progression.	Guo et al.	HNRNPA2B1 can be a useful ESCA prognostic biomarker and therapeutic target.	[61]

Long-chain fatty acids	ESCC	De novo synthesis of strong fatty acids during esophageal cancer cell proliferation and metastasis leads to increased serum long-chain fatty acids.	Jin et al.	Long-chain fatty acids are used as a metabolic sign of tumorigenesis and metastasis of ESCC.	[45]

Linoleic acid	ESCC	Linoleic acid as a metabolite marker	Zhang et al.	Linoleic acid is used to discriminate ESCC and ESD patients and provides helpful reference for clinicians.	[62]

Phosphatidylcholines and choline kinase	ESCC	The key enzyme in the phosphatidylcholine metabolism pathway	Ma et al.	Phosphatidylcholines may be used as novel biomarkers for ESCC.	[63]

PGE2	ESCC	Prostaglandin E2 (PGE2), an active lipid compound derived from arachidonic acid, regulates different stages of the immune response.	Kuo et al.	EP2 expression became an independent factor of overall survival. EP2 overexpression is associated with worse prognosis and correlated positively with T status in ESCC.	[64]

LPE and LPC	Esophageal squamous dysplasia (ESD)/ESCC	Lysophosphatidylethanolamine (LPE) and lysophosphatidylcholine (LPC) serve as a new panel of plasma biomarkers to predict ESCC development.	Zhu et al.	LPE and LPC demonstrated a good diagnostic value.	[8]

FADS1	ESCC	Fatty acid desaturase 1 (FADS1), the rate-limiting enzyme, participates in the desaturation and elongation cascade of polyunsaturated fatty acids to generate long-chain PUFAs.	Du et al.	FADS1 might be a valuable biomarker and potential therapeutic target for ESCC.	[65]

Palmitoleic acid palmitaldehyde isobutyl decanoate	ESCC	A marker board consisting of palmitoleic acid, palmitaldehyde, and isobutyl caprate may be used as an innovative biomarker for the diagnosis and prognosis of ESCC.	Zhu et al.	Palmitoleic acid, palmitaldehyde, and isobutyl decanoate are used as diagnostic biomarkers of ESCC patients.	[47]

Apolipoprotein A1	ESCC	A major component of HDL.	Wang et al.	Apolipoprotein A1 is associated with ESCC patient survival rate.	[66]

CRABP2	ESCC	The CRABP2 gene, a member of the retinoic acid-binding protein family, binds to retinoic acid in the cytoplasm, transports it, and activates the transcription of related genes.	Li et al.	CRABP2 as a suppressor factor is associated with ESCC prognosis.	[67]

**Table 2:** Effective treatment about lipid metabolism-related enzymes and regulatory factor targets in EC.

Compounds	Function	Reference	Clinical trials/inhibitors
ACSS2	Acetyl-CoA synthetase 2 (ACSS2) converts acetate to acetyl-CoA to participate in lipid metabolism and promote whole-body fat storage and utilization.	[71]	Amide-substituted condensed pyridine derivatives [72] tetrazoles [73]
ACC	Acetyl-CoA carboxylases (ACC) are rate-limiting enzymes in de novo fatty acid synthesis, catalyzing acetyl-CoA to form malonyl-CoA.	[74]	Soraphen A piperidinyl derived analogs spiropiperidine derived compounds TOFA ND-630-related compounds Aryl ether derived analogs [68] Haloxyfop sethoxydim Andrimid moiramide B ESP-55016 S-2E CP-640186 [69]
SCDs	Atearoyl-CoA desaturase 1 (SCD1), the enzyme that converts saturated fatty acids to ∆9-monounsaturated fatty acids.	[51]	A939572 MF-438 CVT-11127 CVT-12012 CAY10566 CAY10566 T-3764518 BZ36 SSI-4 SW208108 SW203668 [51]
CPT1	Carnitine palmitoyltransferase I (CPTI) as the key rate-limiting enzyme of FAO facilitates tumor development.	[75]	Etomoxir ST1326 [75]
HMGCR	The third step in the mevalonate pathway, catalyzed by HMGCR, that has been involved in the tumorigenesis of ESCC.	[35, 76]	Statins [77]
FABP1	Fatty acid-binding proteins (FABPs) are intracellular proteins that ingest exogenous long-chain fatty acids (FA) into cells, thereby promoting tumor growth and utilization.	[48, 78]	Niacin derivatives, quinoxaline derivatives, aryl-quinoline derivatives, bicyclicpyridine derivatives, urea derivatives, 1 2,5-dimethyl-[1,2,4]triazolo[1,5-±]pyrimidin-7 (4H)-ones N-(thiophen-2-y)acetamides [79]
CD36	CD36 is a key carrier mediating exogenous uptake of fatty acids and a regulator of ESCC energy sources.	[31, 54]	ABT-510 CVX-O45 ABT-526 ABT-898 CVX-022 3TSR TAX2 ELK-SAHPs [54]
LDL	Low-density lipoprotein is a convenient biomarker and is strongly associated with poor prognosis in esophageal squamous cell carcinoma.	[70]	Anti-PCSK9 antibodies (evolocumab and alirocumab) [80]

**Table 3:** The latest Lipid metabolism drugs with potential clinical use.

Target	Agent	Type	Mechanism	Trial ID or reference
Mitochondrial complex I and metformin ACC	Metformin	ESCC	Metformin can be used as an alternative therapy for chemotherapy- and radiotherapy-refractory esophageal squamous cell carcinoma by inducing cell apoptosis.	[83]
		ESCC	Metformin inhibited the growth and metastasis of ESCC.	[84]

	Metformin with gemcitabine	OSCC	Metformin induces 5-Fu resistance by altering nucleotide metabolism in OSCC.	[85]

	Metformin with cisplatin	ESCC	Metformin combined with chemotherapy can reverse cisplatin resistance by reducing intracellular glutathione levels.	[86]

Natural alkaloid	Berberine	ESCC	By targeting and blocking miR-212, berberine effectively inhibits the invasion and metastasis of ESCC.	[87]
	miR-18b-5p	ESCC	miR-18b-5p regulates de novo lipid synthesis by regulating FASN, ACC1, and SREBP1C and promotes ESCC tumorigenesis and progression.	[33]

Nonsteroidal anti-inflammatory drugs (NSAIDs)	Acetylsalicylic acid (aspirin)	ESCC	Aspirin enhances the therapeutic efficacy of cisplatin in ESCC.	[88]
	Aspirin + statins	OSCC	The combination of aspirin and statin is cost-effective in patients at high risk for progression to esophageal adenocarcinoma.	[89]

HMG-CoA reductase (HMGCR)	Statins (e.g., simvastatin and atorvastatin)	OSCC	The use of statins is associated with a significantly lower incidence of OSCC.	[90]
		ESCC	Atorvastatin inhibits ESCC tumor growth in a PDX model by inhibiting the cAMP and Rap1 signaling pathways.	[91]

Part of carnitine palmitoyltransferase 1 (CPT1)	Carnitine/organic cation transporter novel 2 + Oxaliplatin	ESCC	High expression of OCTN2 promotes the accumulation and cytotoxic activity of oxaliplatin in patients with esophageal cancer, resulting in a reduced risk of recurrence and prolonged survival in EC patients.	[92]

Fatty acid synthase (FASN)	Orlistat (a pancreatic lipase inhibitor developed for obesity treatment), C75, a first-generation synthetic small-molecule inhibitor of FAS, C93, a second-generation small-molecular inhibitor with increased specificity. Previous efforts to treat xenograft cancers with C75	Squamous carcinoma and adenocarcinoma of the esophagus, as well as cases of Barrett's esophagus with varying levels of dysplasia	FAS is expressed at very high levels in esophageal cancer and growth of these cancers can be inhibited by C93. C75 inhibited OSCC proliferation	[39, 81]

Diferuloylmethane	Curcumin	ESCC/OSCC	Curcumin has influences on FAS activity, FAO, and desaturation system. Curcumin may inhibit the proliferation and colony formation of EC according to dose and time.	[93, 94]

Sterol regulatory element-binding proteins (SREBPs)	Fatostatin (4-hydroxytamoxifen, an active metabolite of tamoxifen)	ESCC	Fatostatin significantly inhibited tumorigenesis by downregulating SREBP1 and EMT markers.	[82]

Estrogen receptor (ER) receptor	Natural estrogen (17b-estradiol) selective ER modulators (SERM) tamoxifen and raloxifene	OSCC and Barrett's esophagus	Tamoxifen and raloxifene act as agonists of ER signaling, producing pro-apoptotic and growth-inhibitory effects.	[95]

SQLE inhibitor	siRNA	ESCC	The siRNA significantly inhibited the proliferation and invasion of esophageal cancer cells by regulating the expression of cell cycle and EMT-related proteins.	[96, 97]

## Abbreviations

ESCC: Esophageal squamous cell carcinoma
EAC: Esophageal adenocarcinoma
TME: Tumor microenvironment
EC: Esophageal cancer
BE: Barrett's esophagus
GERD: Complication of gastroesophageal reflux disease
PUFAs: Polyunsaturated fatty acids
NRF2: Nuclear factor erythroid 2 like 2
ACSS2: Acetyl-CoA synthetase short-chain family members 2
FAs: Fatty acids
PI3K/AKT/mTORC1: Phosphoinositide 3-kinase (PI3K)-protein kinase B (AKT)-mechanistic target of rapamycin (mTOR) signaling
ACBP: Acetyl-CoA-binding protein
1-ACBP: 1-Acetyl-CoA-binding protein
B-ACBP: B-Acetyl-CoA-binding protein
PBR: Peripheral *β*-CoA-binding protein
SREBPs: Sterol regulatory element-binding protein
FBP1: Fructose-1,6-biophosphatase
FASN: Fatty acid synthase
ACC1: Acetyl-CoA carboxylase 1
SREBP1C: Sterol regulatory element-binding protein 1C
SCD1: Stearoyl-CoA desaturase 1
SREBP2: Sterol regulatory element-binding protein 2
HMGCR: Hydroxy-3-methylglutaryl-CoA reductase
AMPK: AMP-activated protein kinase
PCNA: Proliferating cell nuclear antigen
NS: Nutritional stress
ACC2: Acetyl-CoA carboxylase 2
CPTI: Carnitine acyltransferase I
TOFA: 5-Tetradecyloxy-2-furoic acid
COX-2: Cyclooxygenases-2
SCDs: Stearoyl coenzyme A desaturated enzymes
ER: Endoplasmic reticulum
SFAs: Saturated fatty acids
PLs: Phospholipids
DAG: Diacylglycerol
TAGs/TGs: Triglycerides
SREBF1: Sterol regulatory element-binding transcription factors 1
EMT: Epithelial-to-mesenchymal transition
MVP: Mevalonate pathway
FAO: Fatty acid oxidation
CPT1A: Carnitine acyltransferase 1A
LDL: Low-density lipoprotein
LRP1B: Low-density lipoprotein receptor-related protein 1B
LRP6: Low-density lipoprotein receptor-related protein 6
EAAs: Essential amino acids
FABPs: Fatty acid-binding proteins
PPARs: Peroxisome proliferator-activated receptors
FABP1: Fatty acid-binding protein 1
FFAs: Free fatty acids
CAAs: Cancer-associated adipocytes
ATGL: Adipose TG lipase
HSL: Hormone-sensitive lipase
MAGL: Monoacylglycerol lipase
DGAT2: Diacylglycerol O-acyltransferase 2
MGAT2: Monoacylglycerol O-acyltransferase 2
SDF-1*α*: Stromal cell-derived factor-1*α*
Rac1: Ras-related C3 botulinum toxin substrate 1
PI3K: Phosphatidylinositol 3-kinase
NF-*κ*B: Nuclear factor-kappa B
JAK/STAT3: Janus kinase/signal transducer and activator of transcription 3
FOXO1: Forkhead Box O1
LPCAT1: Lysophosphatidylcholine acyltransferase 1
SQLE: Squalene monooxygenase
PPAR*α*: Proliferation-activated receptor *α*
M2 macrophages: Anti-inflammatory macrophages
HNF4*α*: Hepatocyte nuclear factor 4 alpha
SIRT1: Sirtuin 1
MDSCs: Myeloid-derived suppressor cells
YAP/TAZ: Yes-associated protein 1/transcriptional coactivator with PDZ-binding motif
NK cells: Natural killer cells
DC cells: Dendritic cells
MCP2: Monocyte chemotactic protein 2
Teff: Effector cells
Treg: Regulatory cells
Tmen: T memory cells
FAS: Fatty acid synthesis
LCFAs: Long-chain fatty acids
CTLA-4: Cytotoxic T-lymphocyte antigen-4
CCL20: C-C motif chemokine ligand 20
IL-10: Interleukin-10
TGF-*β*: Transforming growth factor beta
TRAF6: TNF receptor-associated factor 6
PGC: Peroxisome proliferator-activated receptor-coactivator
PD-1: Programmed cell death protein-1
TILs: Tumor-infiltrating lymphocytes
LCs: Langerhans cell
APCs: Atrial premature complexes
M-MDSCs: Monocytic-myeloid-derived suppressor cells
MSR1: Macrophage receptor 1
FABP-4: Fatty acid-binding protein-4
LDs: Lipid droplets
Wnt5a: Wingless-type protein 5a
ROR2: Receptor tyrosine kinase-like orphan receptor 2
GM-CSF: Granulocyte macrophage colony-stimulating factor
G-CSF: Granulocyte colony-stimulating factor
p-STAT3: Phosphorylated STAT3
p-STAT5: Phosphorylated STAT5
PD-L1: Programmed death-ligand 1
FATP2: Fatty acid transporter 2
OS: Overall survival
IL-6: Interleukin-6
IL-8: Interleukin-8
DCA: Deoxycholic acid
IFN*γ*: Interferon-gamma
CAFs: Cancer-associated fibroblasts
CXCR7: Chemokine receptor type 7
TGF-*β*: Transforming growth factor *β*.
